# Ratcheting up tool innovation in Goffin's cockatoos (*Cacatua goffiniana*): The effect of contextually diverse prior experience

**DOI:** 10.1111/eth.13351

**Published:** 2022-12-19

**Authors:** Paula Ibáñez de Aldecoa, Alice M. I. Auersperg, Andrea S. Griffin, Sabine Tebbich

**Affiliations:** ^1^ Department of Behavioural and Cognitive Biology University of Vienna Vienna Austria; ^2^ Messerli Research Institute, University of Veterinary Medicine Vienna, University of Vienna, Medical University of Vienna Vienna Austria; ^3^ School of Psychology, University of Newcastle Callaghan New South Wales Australia

**Keywords:** behavioural flexibility, parrot cognition, problem‐solving, tool use transfer

## Abstract

The ability to gain information from one situation, acquire new skills and/or perfect existing ones, and subsequently apply them to a new situation is a key element in behavioural flexibility and a hallmark of innovation. A flexible agent is expected to store these skills and apply them to contexts different from that in which learning occurred. Goffin's cockatoos (*Cacatua goffiniana*) are highly innovative parrots renowned for their problem‐solving and tool‐using skills and are thus excellent candidates to study this phenomenon. We hypothesized that birds allowed to use a tool in a larger variety of contingencies would acquire a broader expertise in handling it, facilitating its transfer to new tasks. In our study, we compared the performance of two groups of captive Goffin's cockatoos (*N* = 13): A test group received more diverse learning and motor experiences on multiple applications of a hook‐type tool, while a control group received intensive, total trial‐matched, experience with a single application of the same tool. Then, both groups were tested on two novel tasks to determine whether experience with the tool in multiple contexts would facilitate performance during transfer. While both groups transferred to both novel tasks, group differences in performance were apparent, particularly in the second transfer task, where test birds achieved a higher success rate and reached criteria within fewer trials than control birds. These results provide support for the prediction that experiencing a diverse range of contingencies with a tool appears to allow birds to acquire generalizable knowledge and transferrable skills to tackle an untrained problem more efficiently. In contrast, intensive experience with the tool in a single context might have made control birds less flexible and more fixated on previously learned tool‐dependent instances.

## Graphical abstract

Experimental scheme showing the testing apparatuses used for the test group (upper left panel) and the control group (lower left panel) in phase 1, and for both groups in phase two (right‐hand side panel).

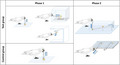

## 1 INTRODUCTION

The ability to acquire information and/or skills from previous experiences and then apply them to new situations is a key element of behavioural flexibility and it is considered to be a hallmark of innovation. While innovating, animals sometimes use new techniques to tackle novel challenges, whereas in other cases they may apply a known or a similar approach to a new purpose (Reader & Laland, 2003). Across the different phases of the innovation process, from discovering an opportunity to establishing an interaction with it, agents can potentially acquire knowledge of varying nature about that opportunity. This knowledge can facilitate solutions to new problems by sorting and applying memory traces of similar experiences (Bobrowicz et al., 2020; Diamond & Bond, 1999; Dukas, 2004; Kamil, 1988; Reader & Laland, 2003; Tebbich et al., 2016). The more general this knowledge is (i.e. less directly connected to a specific opportunity), the larger its scope to be expanded to further unexploited opportunities (Dienes & Perner, 1999). Thus, transfer of knowledge and/or skills (by re‐exploiting a learnt tactic) can be an efficient way to reduce the costs of innovating (e.g. energy and time invested in locating and inspecting a food source; Klump et al., 2015), eventually allowing innovations to extend beyond the initial context (Tebbich et al., 2016). In this study, we aimed to manipulate the diversity of experiences that subjects had prior to encountering a novel problem to test how the resulting variation in knowledge (specific or broad) may affect performance during transfer.

Tool use tasks seem particularly suited to this purpose, as the same tools can often be applied in multiple contexts: innovations that incorporate objects (and tools in particular) can potentially have a wider applicability (Tebbich et al., 2016). The concept of “tooling” was proposed by Fragaszy and Mangalam (2018) to delineate the different stages of tool use and the tool‐object interactions they entail. While tooling, toolers (1) perceive the prospect of producing an effect upon a target with a grasped object, (2) transform their body into a body + object system and (3) create a mechanical interface with said target by establishing spatial relation(s) between the target and the tool. As a result, when handling a tool to solve a problem, animals can acquire knowledge about its functional features and affordances such as its shape, size and weight, by incorporating actions with objects and managing the tool–target relations established (Auersperg, Gajdon, & von Bayern, 2012; Call, 2012; Diamond & Bond, 1999; Fragaszy & Mangalam, 2018; Griffin & Guez, 2014). They could then potentially use this information to assist in future novel problems and innovative foraging that require the use of tools (Auersperg, 2015; Diamond & Bond, 1999; Lambert et al., 2017; Taylor, Hunt, et al., 2009; Taylor, Roberts, et al., 2009). Transfer of such acquired tool‐related proficiency also requires behavioural adjustments, such as refining the motor output and combinations of different motor actions (Griffin et al., 2014; Griffin & Guez, 2014; Guez & Griffin, 2016; Tebbich et al., 2016). Therefore, transfer in tool‐using contexts can be studied particularly well in species with a high capacity to innovate in technically challenging situations and who have a wide motor repertoire.

We tested Goffin's cockatoos (*Cacatua goffiniana*, henceforth Goffins), a species that deploys numerous foraging innovations and which is renowned for its extraordinary examples of flexible tool use in captivity (Auersperg et al., 2013; Auersperg, Gajdon, & von Bayern, 2012; Auersperg, Szabo, et al., 2014; Auersperg, van Horik, et al., 2014; Auersperg, von Bayern, et al., 2014) and, as we recently learned, also in the wild (O'Hara et al., 2019, 2021; Osuna‐Mascaró & Auersperg, 2018). This trait seems to be facilitated by their beak–foot motor coordination to hold and control a tool, which, especially in parrots, permits a great diversity of manipulation alternatives (Mettke‐Hofmann et al., 2002). Their feeding opportunism and extractive foraging behaviour in the wild may make it adaptive to innovate based on experience when faced with similar but slightly different problems. A recent study (O'Hara et al., 2021) showed that Goffins flexibly implement the use of a tool depending on the characteristics of the target encountered, a complex technique that requires careful consideration of two different concurrent spatial relations: holding the food with the foot and orienting the tool for insertion with the beak. New evidence also indicates that Goffins can selectively recombine relevant previous experiences to solve novel problems that partially match (either functionally or perceptually), by relying on flexible memory functions (Bobrowicz et al., 2021). This faculty had already been demonstrated in great apes: Ebel and Call (2018) found that gorillas (*Gorilla gorilla*), bonobos (*Pan paniscus*), chimpanzees (*Pan troglodytes*) and orangutans (*Pongo abelii*) exposed to an empty apparatus before the test (with a baited apparatus) gained information that was later used to solve a problem‐solving task more efficiently, while Bobrowicz et al. (2020) showed that chimpanzees and orangutans attend to relevant aspects of a problem and draw on those past experiences to assist current problem‐solving.

**FIGURE 1:**
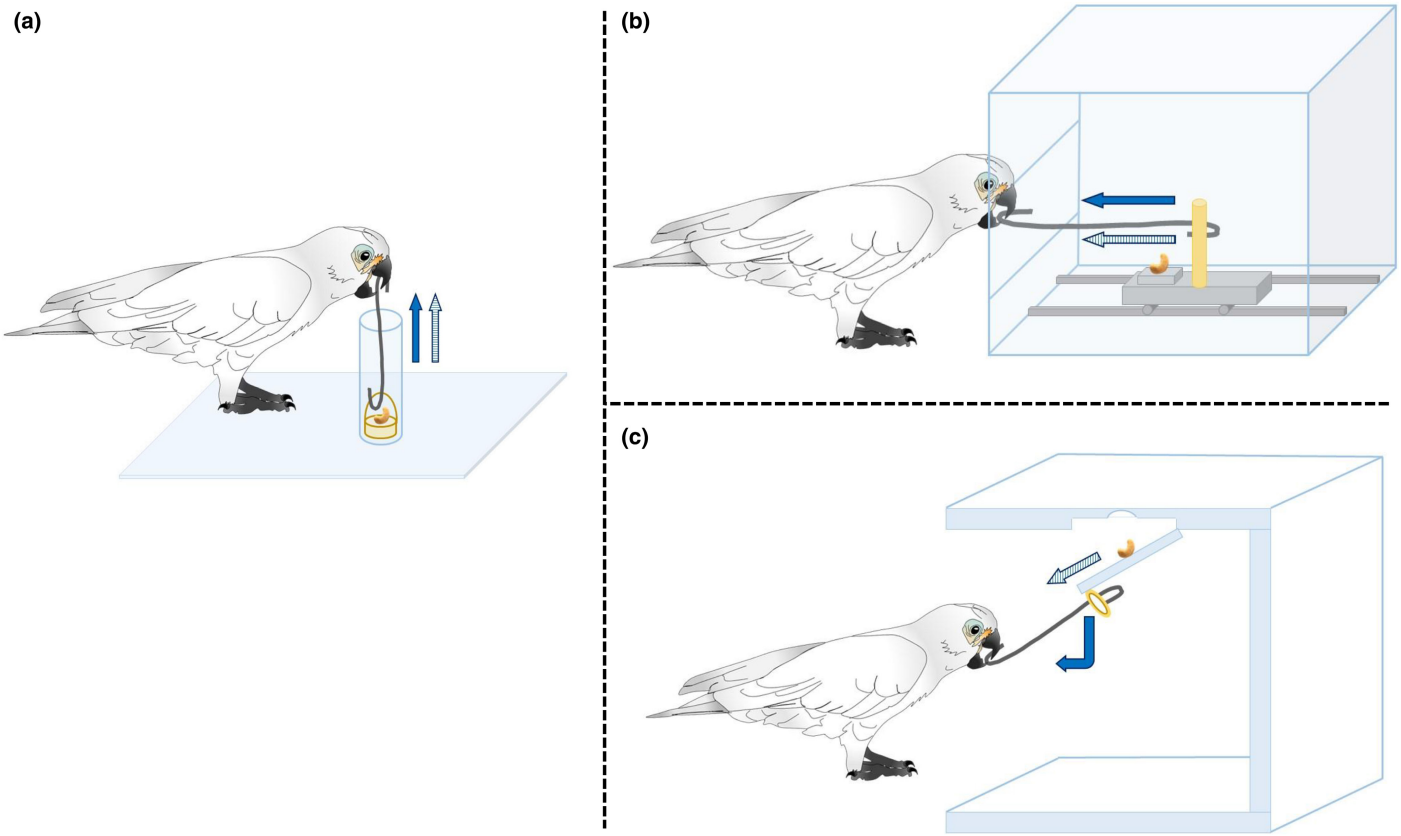
Apparatuses used for phase 1. (a) Basket: The reward is obtained by probing the hook through the transparent tube and inserting it through the basket's handle, then lifting it up. (b) Skate: The reward is obtained by passing the hook through the front window (left side of the drawing) and grabbing the protruding stick on the skate with the hook, then pulling it out. (c) Trap: The reward is obtained by inclining the hook upwards and grasping the ring with it, then pulling the trap down to open. Elements in yellow indicate target in the apparatus. Dark‐coloured arrows denote the movement displayed by the bird and light‐coloured arrows reflect the directionality of the reward following bird's movement.

Here, we investigated whether previously acquired experiences about a tool and its possible applications can be transferred to solve a new, untrained problem. For this, we provided birds with a single tool (a hook) that could be used for solving different tasks by adapting a combination of learned motor patterns. In phase 1 of the experiment, a test group was given experience with three tasks where the tool was freely moving. Each task required a different pulling motion with the tool, however. Each one also required motor coordinating a different tool–target interaction: (1) inserting the hook through a handle from above and pulling *upwards* (basket task), (2) inserting the hook through a window from the front and pulling horizontally *towards the self* (skate task) and (3) inserting the hook into a vertically oriented ring from below and pulling *downwards* (trap task; see Figure 1a–c). In contrast, the control group received intensive, total trial‐matched experience with the same tool in only one (basket/pull *up*) task. Using a similar methodological approach to ours (by controlling and varying pretesting experience but focusing on how groups differed in their knowledge of the task rather than in that of the tool), von Bayern et al. (2009) found that New Caledonian crows (*Corvus moneduloides*) can innovate after acquaintance with the functional properties of a task. Similarly, a study in great‐tailed grackles (*Quiscalus mexicanus*) suggests that behavioural flexibility can be shaped through experimental manipulation using reversal learning, and lead to an improvement of problem‐solving in a new context, namely a multi‐access box (Logan et al., 2022, preprint).

**FIGURE 2:**
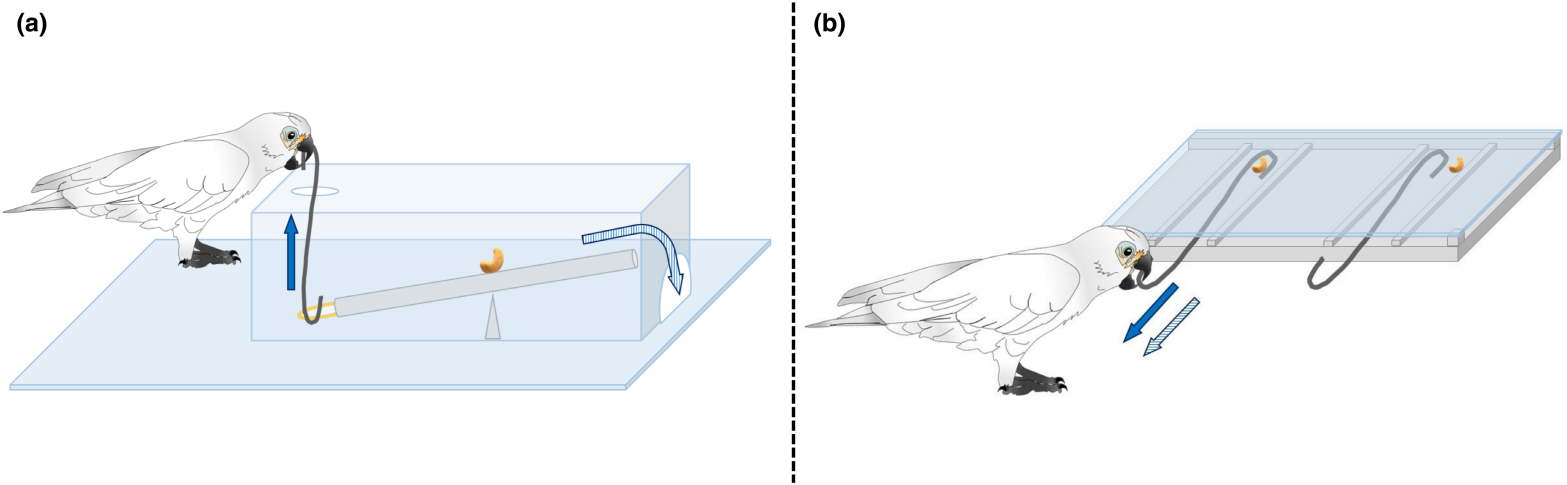
Apparatuses used for phase 2. (a) Seesaw task: The reward is obtained by actively inserting the hook through a hole located on the top of the box, grasping a lever (target element, coloured in yellow) at the end of the seesaw and pulling it upwards. (b) Cane task: The reward is obtained by pulling the pre‐inserted functional hook towards the bird. Dark‐coloured arrows denote the movement displayed by the bird and light‐coloured arrows reflect the directionality of the reward following bird's movement.

In phase 2 of the experiment, we measured the effect of the aforementioned differential training on performance in a new task, the seesaw (see Figure 2a). Two main features made this task novel: (1) the tool–target interaction differed from that of any apparatus presented in phase 1 (the hook was used to hoist a horizontally oriented lever located on the side of the seesaw), and (2) lifting the seesaw up caused the reward to slide away from, rather than towards, the self and be delivered at the opposite side of the apparatus. After the birds had completed testing with the seesaw task (while data collection was still on going and prior to analysis), it become apparent that, despite the very distinct features mentioned above, inserting the tool from above and pulling it upwards were actions similar to those used in the basket task, with which both groups had had prior experience. Therefore, we decided to conduct a follow‐up experiment by testing the birds on a second task that was perceptually even more different, so as to rule out the role of the motor actions. In our second transfer task (the cane; see Figure 2b), the contribution of the motor component was strongly reduced, as the tool was not freely manoeuvrable (i.e. it was already pre‐inserted in the apparatus). In addition, in contrast to the seesaw, using the tool made direct contact with the reward (without mediation of a target). Hence, the birds had only to pay attention to the functional relationship between the tool and the reward rather than negotiate a tool to target contact to indirectly retrieve the reward. Furthermore, prior work with the cane task (Darwin's finches: Teschke et al., 2011) and other similar transfer tasks (Corvids: Bird & Emery, 2009; Emery & Clayton, 2009) allowed for performance of Goffins to be compared with other bird taxa.

We predicted that providing the test group with the opportunity to use the tool in a larger variety of contingencies in phase 1 (three different problem‐solving situations as opposed to one) would allow them to explore the affordances of the hook and acquire more diverse expertise in handling it, increasing their tool‐related knowledge and ultimately facilitating a more skilful solving competency in phase 2 (Griffin et al., 2013; Griffin & Guez, 2014; von Bayern et al., 2009).

## 2 MATERIALS AND METHODS

### 2.1 Subjects

Five female and eight male adult captive‐reared Goffin's cockatoos (6–10 years of age) participated in the study. Subjects were assigned to a group at random by using the *sample* function in R, after establishing an equal sex distribution as a grouping criterion. The composition of the groups, once balancing the number of males and females in each, was: Control (*n* = 6): two females (Olympia, Moneypenny) and four males (Kiwi, Konrad, Muki, Zozo); and Test (*n* = 7): three females (Fini, Heidi, Mayday) and four males (Dolittle, Figaro, Muppet, Pipin). Although age was not considered when forming the groups, age differences (values at the time of testing, in 2019) were not significant (Test = 9.85 ± 1.78; Control = 8.83 ± 0.41; *t* = 0.246; *p* = .813). For additional subjects' information and details on housing conditions see Table S1. All subjects received a diet consisting of a source of carbohydrates mixed with fruits, bird food pellets, powdered vitamin supplements and seeds, as well as ad libitum drinking water. Participation was voluntary and birds were not food deprived prior to the experiment. Subjects were in semi‐free housing conditions, with access to indoor and outdoor aviaries, without social isolation but visually isolated during the testing sessions, when the experimental subject was briefly separated from the group to avoid social learning. None of the toys provided as environmental enrichment resembled the experimental apparatuses or the tool. Data collection took place between March and September 2019 at the Goffin Lab Goldegg, located in Lower Austria.

#### 2.1.1 Experimental histories and STRANGEness of our test sample

To acknowledge the potential influence of subjects past experience in our study, following the recommendations made by the STRANGE framework (Webster & Rutz, 2020), we declare that all birds had previously participated in a number of experiments on tool use and other technical problem‐solving tasks involving the use of acrylic glass apparatuses (for some examples see Auersperg, Szabo, et al., 2012; Habl & Auersperg, 2017; Laumer et al., 2016, 2017). However, although birds had a different hatching date (see Table S1), tool use experiments in the Goffin Lab only started in 2012. This means that age does not equate to experimental history, since all subjects started taking part in experiments at the same time; hence, older birds did not necessarily accumulate more testing experience. All tasks (with the exception of the basket task, which was previously used in Laumer et al., 2017) were novel to all individuals of both groups. In that study, which used a bendable hook‐shaped tool (a pipe cleaner), only Fini and Moneypenny learned to consistently make and use this type of tool.

### 2.2 Apparatuses and procedures

All tasks required the use of a hooked tool to gain access to the reward. At the beginning of the experiment, all birds had equal prior experience in using the tool, as it was used in the training stage of a previous study published by our group (Laumer et al., 2017). The tool was a 7‐cm‐long S‐shaped hook made of rigid stainless steel, a material that ensured that no modifications could be made by the birds. The food reward (a 1/8th piece of a cashew nut, which birds only received when participating in the experiment) was visible yet unreachable without the tool, and it was located inside one of five apparatuses made of 0.5‐mm thick Plexiglas and plywood. Apparatuses were always baited out of the bird's sight. At the beginning of the experiment, there was a 2‐day habituation period for each task separately. First, habituation was undertaken in a group setting by placing the unbaited apparatus without the tool in the aviary until all birds had approached it voluntarily. This habituation phase ended when all subjects had contacted the apparatus or fed in its proximity at least once. Next, habituation was undertaken in an individual setting by presenting each subject with the unbaited apparatus without a tool and allowing the bird to inspect it. In both settings (collective and individual), subjects were encouraged to approach the apparatus by spreading sunflower seeds around it. Habituation to the hook was also conducted prior to testing: each bird had to pick up the tool from the table and exchange it for a seed with the experimenter on five consecutive trials during three sessions.

In the experimental sessions, the experimenter remained in the testing room, wearing mirrored sunglasses and looking down with hands off the table to avoid interfering with the bird's performance or providing any indirect cues. Testing consisted of two phases: in phase 1 a test group and a control group received different experiences and in phase 2 both groups received two new tasks. Throughout both phases, all birds were tested for two sessions a week, where the order of subject's testing was randomised within sessions to prevent the same individuals from being tested always at the same time of the day. All trials were video recorded (HD‐Camcorder HC‐V160, Panasonic), and latencies were measured using a stopwatch.

#### 2.2.1 Phase 1

Phase 1 differed between groups: while birds in the test group gained a broader diversity of experiences on three different tasks (namely the basket, the skate and the trap), control group received intensive experience on the basket task only. To solve the problem and obtain the reward, each task required birds to perform a motor sequence that also differed in the direction of pulling with respect to gravity: in the basket, birds had to pull the hook *upwards* (see Figure 1a), whereas in the skate they had to pull the hook *towards the self* (see Figure 1b). Finally, the trap required birds to pull the hook *downwards* (Figure 1c). Similarly, the tool–target interaction differed for each task: lift a handle upwards in the basket, drag a mobile miniwagon horizontally in the skate and pull a ring downwards in the trap. Training with these tasks in phase 1 could enhance flexibility for transfer in phase 2 because the motor action needed and the position of the bird's body with respect to the tool were different for each apparatus; rapid motor adjustments were required to decouple the learnt pattern from a specific problem and permit its flexible adaptation to a new task (Fragaszy & Mangalam, 2018; Griffin et al., 2014): from leaning the head down in the basket, to bending the upper body forward in the skate or stretching the neck and tilting the head slightly up in the trap (see Figure 1a–c). The order of presentation of these three tasks was randomized across subjects in the test group as shown in Table 1.

**TABLE 1:** Experiment timeline for the test group (top three rows) and control group (bottom row) in phase 1 (left column) and phase 2 (right column)

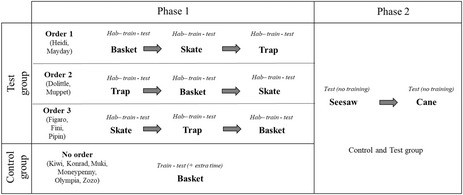

*Note*: Phase 1: for the Test group, the three possible orders of presentation are shown (order 1, 2 and 3), indicating between brackets the names of the subjects tested on each order. For each of the three tasks (basket, skate and trap), the experiment sequence was habituation (“*hab*”) > experience or training (“*train*”) > testing (“*test*”) > extra time (applicable only for some subjects of the control group). Phase 2: all subjects were equally tested on the seesaw task and the cane task; unlike in phase 1, in phase 2 testing commenced directly, without previous training.

Phase 1 consisted of the aforementioned habituation followed by a training stage and a testing stage (12 trials per session). A detailed summary of the testing scheme is shown in Table 1. During training and testing, birds were given an unlimited amount of time per trial to solve the task, but if they did not interact with the tool or the apparatus for 15 min, the trial was terminated. The purpose of the training stage was for the birds to learn the tool functioning in a particular task. To aid slowly engaging individuals (e.g. those who did not manage to grab the tool and direct it towards the target at this stage), we adapted our experimental protocol by providing step‐by‐step shaping (i.e. the experimenter demonstrated how to handle the tool rightfully and how to insert it in the apparatus) until such individuals could solve the task autonomously in five consecutive trials. The success criterion for the training stage was solving correctly 80% of trials within a session. Once this criterion was met, birds moved on to the testing stage, where the goal was to put into practice the skills acquired in the training stage without receiving any help from the experimenter. In the testing stage, birds had to solve 100% of trials per session in two consecutive sessions to ensure that they had completely mastered the use of the tool in a given apparatus before proceeding to the next one. In the case of the control group, subjects continued being tested on the basket task even after reaching solving criterion on the testing stage, to ensure an equivalent overall number of trials as the test group and a comparable amount of experience time with the hook. The purpose of this extensive training was to try to guarantee that all birds achieved the same level of competence before moving on to phase 2. These additional sessions were labelled as “extra time.” The number of sessions required per bird in each task can be found in Table S5 (test group) and Table S6 (control group) of the SI.

#### 2.2.2 Phase 2

In phase 2, both groups were tested on two novel, untrained tasks: the seesaw and the cane. In contrast to phase 1, birds commenced phase 2 testing immediately (i.e. without previous training). In the seesaw task, birds had to first insert the hook through a hole located in the top of the covering box, grasp a lever at the end of a seesaw and pull it upwards. This motor action lifted the seesaw, making the reward slide away from the self, down to a location where it could be collected by the bird (Figure 2a). Hence, the seesaw task differed from the other tasks in the immediate effect following the tool's movement: while in the rest of the tasks the hook always moved the reward closer to the bird, in the seesaw task its effect was to push the reward away before it became accessible to the bird elsewhere. Another crucial characteristic was that the target element in this apparatus (lever) had a horizontal orientation, in contrast to the vertical orientation of the target element in the basket (handle). Birds were administered a maximum of three 12‐trial sessions for this task, and success criterion was solving 80% of the trials in one session.

Once testing with the seesaw had concluded, each bird was tested on a second task that lacked any common features with previously presented tasks. The cane task was a two‐choice task in which two identical‐looking baited hooks were laid out parallel to each other on a wooden platform covered with a transparent Plexiglas sheet (Figure 2b). Each hook was fitted into a groove to prevent it from flipping and to ensure that it could only be pulled straight. A similar version of this task was used in a previous study with Darwin's finches (Teschke et al., 2011). In each trial only one of the hooks was functional (i.e. it led to obtain food when pulled). Which hook was made functional was counterbalanced across trials. If a subject picked the functional hook, it could access the reward; if the hook picked was the non‐functional, the apparatus was removed from the bird's reach, ending the trial. Most importantly, the main aspect distinguishing this task from the other tasks used in our study was that the hooks were already pre‐inserted in the apparatus, hence not requiring birds to apply any complex motor action other than picking a hook and slightly drag it towards themselves. To make the present study results comparable to those from the Darwin's finches' study (where birds received a maximum of 140 trials and had to make 15 or more correct choices within two consecutive blocks of 10 trials), our Goffins received a maximum of ten 12‐trial sessions in the cane task. Given that this was a two‐choice task, we also applied a more stringent solving criterion to be more confident that success was not attributable to chance. The solving criterion was set at 80% of correct trials per session in two consecutive sessions. Testing in the cane task ended either when criterion was reached or once the bird had received 10 sessions.

In both tasks, a trial started as soon as the subject touched the tool and lasted either for 5 min or until the bird solved the task. In contrast to phase 1, phase 2 involved no shaping or feedback to the birds. The variables measured (values between brackets) were *session number* (1–3 in the seesaw task; 1–10 in the cane task), *trial number* (1–12 in both tasks), *solved* (0 = not solved; 1 = solved) and *time to solve* each trial (0–300 s).

### 2.3 Statistical analyses

#### 2.3.1 Success probability in the seesaw task (model 1) and the cane task (model 2)

All statistical analyses were performed in RStudio (version 4.0.3, R Core Team, 2020). To analyse the effect of *sex*, *group* and *session number* on the *probability to solve a trial* in phase 2, we fitted two (one model per task) generalized linear mixed models (*glmer* function) with binomial error structure and *logit link* function of the package lme4 (version 1.1–25, McCullagh & Nelder, 1989). The response variable *probability to solve a trial* was a matrix with two columns: the first one grouping number of solved trials per individual and session, the second one with the unsolved trials (i.e. trial performance was aggregated at the session level). We included an interaction between *group* and *session number*, and we further included *individual* as random effect. We also z‐transformed *session number* to a mean of zero and a standard deviation of one to achieve easier interpretable estimates while avoiding potential convergence issues (Schielzeth, 2010). We used Wald's *z*‐approximation (Field, 2005) to infer about the significance of individual effects.

We divided the original data set into two subsets, namely the seesaw (model 1) and the cane (model 2) to fit two separate models, one for each of the tasks in phase 2. The number of observations in these subsets was 300 for model 1 and 1116 for model 2. In model 1, we fitted the full model with a control structure and added the optimizing algorithm *bobyqa* (Bound Optimization BY Quadratic Approximation; Powell, 2009), aiming to maximize convergence probability. In model 2, the full model suggested that the correlation among random slope of intercept for session number (a parameter which is recommended to routinely include; see Barr et al., 2013) was estimated to be essentially 0 (i.e. it was not appropriately identifiable due to the complexity of the model and the small sample size; Matuschek et al., 2017). Therefore, we fitted a full model without the correlation parameter, which showed an almost identical log‐likelihood and no substantial differences with respect to the fixed effects, and we used this model for further inferences. The distribution of individual‐specific deviations from the common intercept and slopes was symmetrical. The full model did not present any collinearity issues, and it was stable. The null model, lacking *group*, was fitted separately for model 1 and 2. In both cases, full‐null model comparisons were conducted using a χ^2^ test. To infer the significance of the terms involved in the interaction, we conducted likelihood ratio tests by dropping one term at a time from the full model and comparing it to the null model, whereby the *p*‐values obtained are considered significant only if the comparison reveals significance (Mundry, 2014). We determined the effect size for the full as compared to the null model with the function *r.squaredGLMM* of the package *MuMIn* (Barton, 2009), and confidence intervals of fitted values at 95% using the function *confint* of the package *base*. To rule out collinearity issues, we calculated Variance Inflation Factors (VIF; Field, 2005), with the function *vif* of the package *car* (version 3.0–10, Fox & Weisberg, 2011), applied to a model lacking the interaction and the random effects.

#### 2.3.2 Time to solve in the seesaw task (model 3) and the cane task (model 4)

Furthermore, we checked whether group's performance would differ throughout testing in both tasks, that is that groups would differ in time to solve a trial across sessions and across trials within session. To address this question, we performed two mixed effects Cox regressions (also known as survival analysis), one for each task separately, using the previously generated subsets (*seesaw* for model 3 and *cane* for model 4). The number of observations was 468 for model 3 and 1560 for model 4. In these models, the response variable time to event was measured as “time” in seconds needed to solve each trial in phase 2, and *solved* >0 was used as the argument for “event.” We fitted the models using the function *coxme* of the package *coxme* (Therneau, 2020) including interaction terms between *group*, *trial ID*, and *session number*, a fixed effect of *sex*, and a random effect of *individual*. The 3‐way interaction, formulated as group*trial ID*session number, included all the 2‐way interactions and the main effects it encompassed. We created the survival object with the function *surv* of the package *survminer* (Kassambara et al., 2020). Prior to fitting the models, we *z*‐transformed *session number* and *trial ID* to a mean of zero and a standard deviation of one. To control for cryptic multiple testing (Forstmeier & Schielzeth, 2011), we performed full‐null model comparisons between the full models (comprising *group* and all the interactions this term was involved in) and the null models (identical to the full models with respect to the random effects structure but lacking *group* as a main effect but also without all the 3‐ and 2‐way interactions that *group* was involved in); comparisons were conducted by using a likelihood ratio test (Dobson, 2002). We aimed to assess model stability (which reveals how much the estimated coefficients change when individual cases are excluded) by dropping datapoints, one at a time, then fitting the full model to each of these subsets and comparing the range of model estimates obtained with those obtained for the full data set. Model stability could not be determined due to the complexity of the models. However, although this might slightly influence the confidence that we can have on the conclusions drawn from our models, we did not expect their validity to be compromised, as we took all the necessary steps prior to fitting the models to ensure their reliability.

## 3 RESULTS

### 3.1 Qualitative description of performance in the seesaw task and the cane task

In the seesaw task, groups did not differ in the number of sessions required to reach criterion (mean ± SD = test: 1.85 ± 0.89 vs. control: 2 ± 1.1; range = 1–3; *t*_(11)_ = −1.869; *p* = .092). Although the same number of test subjects as control subjects reached criterion in session 1 (test group (*n* = 3): Figaro, Fini, Muppet; control group (*n* = 3): Kiwi, Konrad, Zozo), across all 3 sessions the test group contained a higher proportion of successful individuals (100% vs. 66%). This was due to two individuals from the control group (Muki and Olympia) not reaching criterion for this task. No group differences were detected in the average number of trials solved per session (test group: 11 ± 1.41; control group: 11 ± 1; *t*_(11)_ = −0.582; *p* = .577): the best‐performing test group birds (Figaro and Muppet) solved as many trials per session (12 trials) as the best‐performing control group bird (Kiwi). The average time to solve a trial in session 1 in the control group was almost double than that of the test group (125 s vs. 67 s), with Figaro (test group) being the fastest (27.4 s) and Olympia (control group) the slowest bird overall (260.6 s). For detailed information on learning criterion and solving accuracy, the reader is referred to Tables S3, S7b,d, S9b and S10b of the SI.

In the cane task, groups differed in the number of sessions needed: individuals in the test group required between 4 and 7 sessions to reach criterion (mean ± SD = 5.57 ± 1.27), while the control group required between 7 and 10 sessions (9 ± 1.26; *t*_(11)_ = −4.227; *p* = .001). The fastest subjects in the test group (number of sessions in brackets) were Dolittle and Fini (4), followed by Pipin (5), Muppet and Figaro (6), and Mayday and Heidi (7). The fastest bird in the control group (Kiwi) needed as many sessions (7) as the slowest birds in the test group (Mayday and Heidi). Kiwi was followed closely by Zozo (8) and Konrad (9) in the control group. The same three individuals from the control group (Muki, Olympia and Moneypenny) that did not reach criterion for the seesaw task also did not reach it for the cane task. Groups also differed in their solving accuracy in this task, with the test group having a higher average number of solved trials per session (8.42 ± 0.45) compared with the control group (7.76 ± 0.75; *t*_(11)_ = 2.645; *p* = .024). Muppet (test group) was the bird with the highest average number of solved trials (9.2) whereas Olympia (control group) had the lowest (6.7). There were no differences in the average time to solve a trial in session 1 between the test and the control group (6.26 vs. 10.78 s; *t*_(11)_ = −0.992; *p* = .351), with Fini (test group) being the fastest (2.4 s) and Muki (control group) the slowest (69.7 s) of birds overall. For detailed information on learning criterion and solving accuracy, the reader is referred to Tables S2, S7a,c, S9a and S10a of the SI.

### 3.2 Success in the seesaw task (model 1) and in the cane task (model 2)

In the seesaw task, the full‐null model (model 1) comparison indicated that no group differences were found in the probability to solve a trial across sessions (*χ* ^2^_(2)_ = 1.545; *p* = .219; see Table S4 of the SI). However, the fact that both groups' performance was above 50% of accuracy already from session 1 (test group: 78.3%; control group: 62.5%) indicates that they both indeed transferred immediately (see Table S9b of the SI).

**FIGURE 3:**
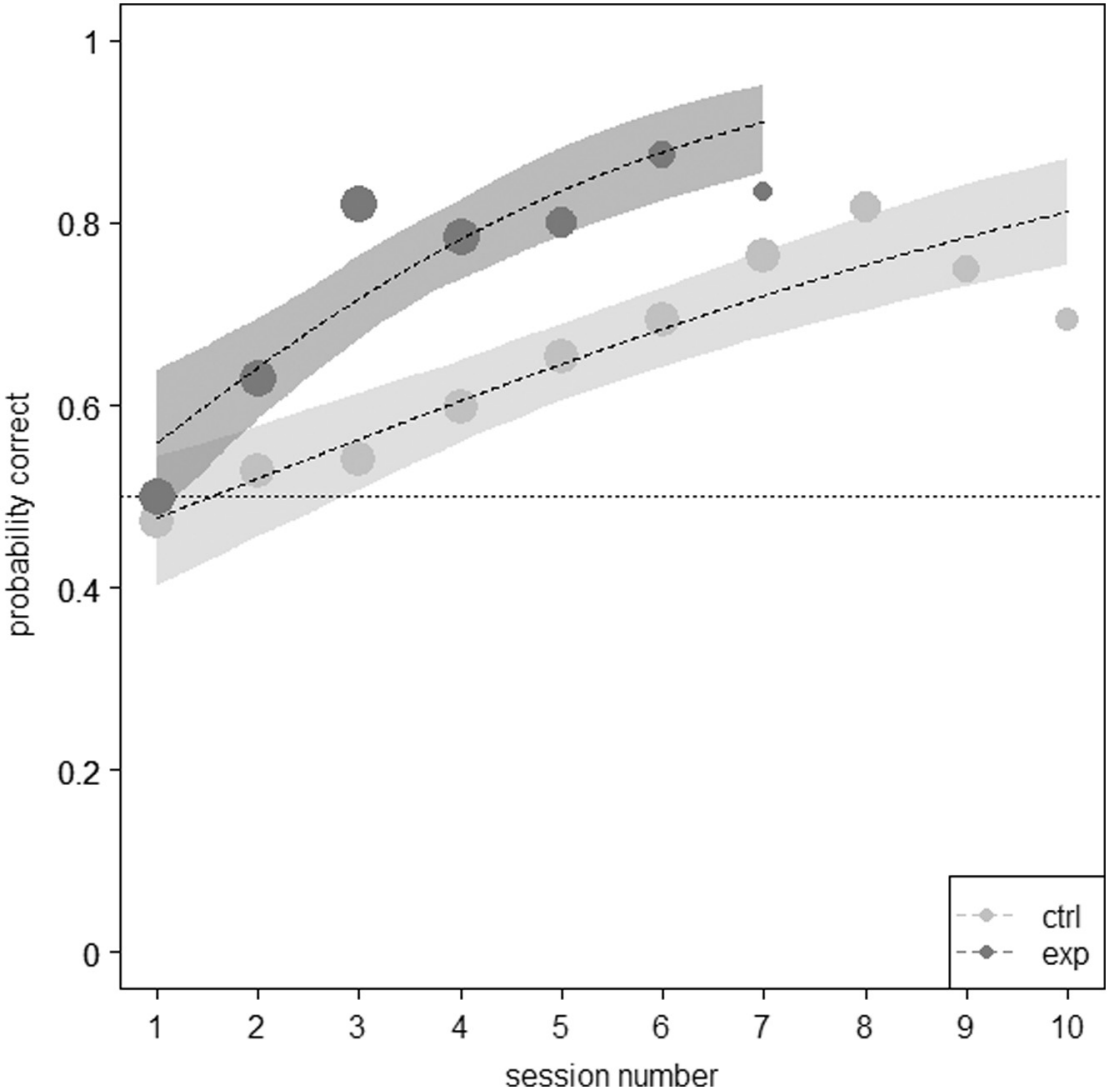
Probability of a trial being solved in the cane task as a function of session number. Performance of control group (ctrl) is shown in light grey, and test group (exp) is depicted in dark grey. Point area is proportional to the number of subjects that participated on each session (i.e. areas have different sizes because of some individuals reaching solving criterion earlier than others). Shaded line corresponds to 95% confidence intervals. Horizontal dotted line reflects performance at chance level. The plot shows the model with *sex* centred and 1000 bootstraps

In the cane task, the full model (model 2) detected a significant interaction between *group* and *session number* (*F*_(1, 2)_ = 5.631; *p* = .018; see Table 2), indicating that groups differed with respect to their probability of solving a trial across sessions, being this higher for the test group. The full‐null model comparison revealed an effect of *group* on the number of solved trials per *session number* (*χ* ^2^_(2)_ = 19.631; *p* < .001; *R* ^2^_fixed + random effects_ = .475). Hence, while transfer occurred in both groups, it was not immediate (i.e. it did not occur from session 1), and the test group solved the task faster (as reflected by a steeper learning curve; see Figure 3).

**TABLE 2:** Coefficients for fixed effects of the glmer (cane task, model 2, full model)

	Estimate	SE	*p*‐value	Lower CI	Upper CI
Intercept	0.569	0.108	**<.001** *	0.347	0.804
Group	0.917	0.176	**<.001** *	0.563	1.277
Session nr.	0.435	0.082	**<.001** *	0.276	0.597
Sex	−0.191	0.148	.196	−0.500	0.118
Group:Session nr.	0.438	0.185	**.018** *	0.080	0.807

*Note*: Reference values for *group* and *sex* are “control” and “male,” respectively; *Session number* was *z*‐transformed; original mean ± SD = 3.805 ± 2.512.

Abbreviations: nr., number; SE:,standard error; CI, 95% confidence intervals; (:), interaction term.

* Significant *p*‐values at *α* = 0.05 are shown in bold.

### 3.3 Time to solve a trial in the seesaw task (model 3) and the cane task (model 4)

**FIGURE 4:**
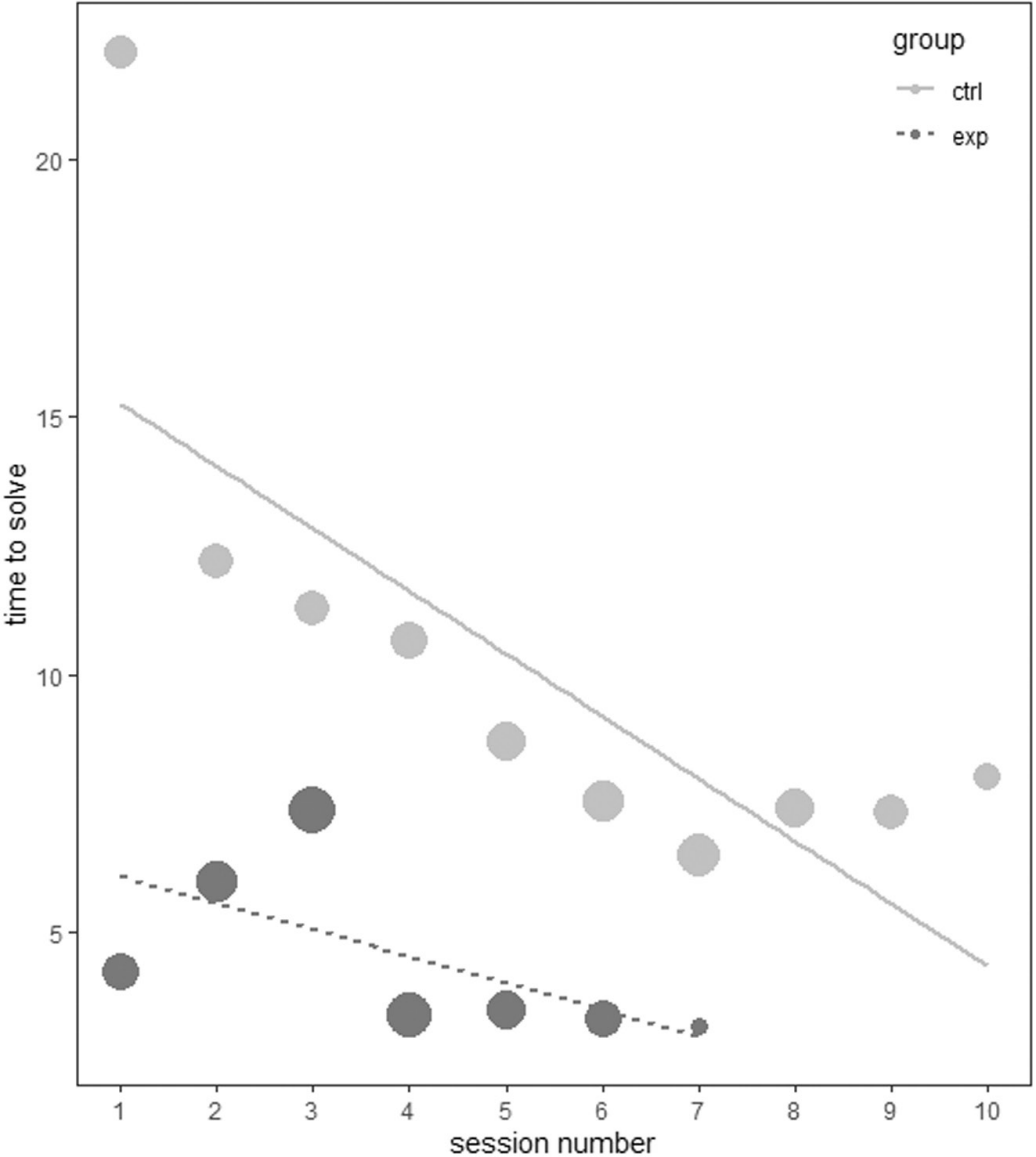
Time to solve a trial (in seconds) in the cane task as a function of session number. Performance of control group (ctrl) is shown in light grey with a solid line, and test group (exp) is depicted in dark grey with a dashed line. Point area corresponds to the number of solved trials per session across all subjects of the same group; lines represent the prediction made by the fitted model. Values on the *y*‐axis are in seconds.

In the seesaw task, we did not find a significant effect of *group* on *time to solve a trial across sessions* (*χ* ^2^_(4)_ = 2.925; *p* = .571; see Table S8 of the SI), suggesting that both groups' performance remained equally fast throughout testing. In the cane task, on the contrary, the full‐null model comparison revealed a significant effect of *group* on *time to solve a trial across sessions* (*χ* ^2^_(4)_ = 29.104; *p* < .001.; see Table 3; see Figure 4), indicating that individuals from the test group solved a trial significantly faster than individuals from the control group across sessions. This meant that, not only did the test group learn the task faster but they also showed more improvement in their solving accuracy along testing.

**TABLE 3:** Coefficients for fixed effects of the Cox regression (cane task, model 4)

	Coef.	Exp.	SE	*p*‐value	Lower CI	Upper CI
Group	1.801	6.057	0.691	**.009** *	0.446	3.156
Trial nr.	0.319	1.377	0.055	**<.001** *	0.211	0.428
Session nr.	1.135	3.110	0.122		0.894	1.375
Sex	0.161	1.175	0.700	.83	−1.211	1.534
Group:Trial nr.:Session nr.	−0.124	0.884	0.086	**<.001** *	−0.292	0.044

*Note*: Reference values for *group* and *sex* are “control” and “male,” respectively; *Session number* and *trial number* were z‐transformed; original mean ± standard deviation = 5.5 ± 2.87 and 6.5 ± 3.45, respectively.

Abbreviations: nr., number; SE, standard error; CI, 95% confidence intervals; Coef., coefficients; Exp., exponentiated coefficients; :, interaction term.

* Significant *p*‐values at *α* = 0.05 are shown in bold.

A summary of performance of both groups in both tasks is shown in Table 4.

**TABLE 4:** Summary of group differences in the seesaw task and the cane task

Variable	Seesaw task	Cane task
Number of sessions to reach criterion	Test = Control	Test < Control
Proportion of successful individuals	Test > Control	Test > Control
Number of trials solved per session	Test = Control	Test > Control
Time to solve a trial in session 1	Test < Control	Test < Control
Probability to solve a trial across sessions	Test = Control	Test > Control
Time to solve a trial across sessions	Test = Control	Test < Control

Abbreviations: =, groups did not differ in their performance on the corresponding variable; <, first group (test) had a lower score in the corresponding variable; >, first group (test) had a higher score in the corresponding variable.

## 4 DISCUSSION

We studied tool transfer in Goffin's cockatoo by investigating whether the capacity to apply previously acquired information about a tool and its use from one context to a new one changes as a function of contextually diverse prior experience with the same tool. Our findings indicate that Goffin's cockatoos can transfer knowledge and derived skilfulness about a hooked tool to new contexts in which they received no training and different from those in which learning occurred and that individuals who received broader experience perform better in transfer.

Transfer was originally planned and tested on one task only, the seesaw. We initially considered this task to be sufficiently new to all birds for two major reasons: First, the application of the tool onto the target element in the seesaw (lever) did not allow to retrieve the reward immediately, as it was delivered to the opposite side of the apparatus once the seesaw was lifted up; and second, the orientation of said lever was horizontal, as opposed to the vertically oriented target in the basket (handle). However, it became apparent when most birds from both groups transferred almost immediately that despite the design modifications, the seesaw task shared a similarity in the sequence of movements required to find a solution with the basket (which was common for both groups in phase 1). This resemblance affected in particular the motor operating, as solving the seesaw involved some steps in which both groups had gained experience (e.g. inserting the hook vertically and pulling upwards). This could therefore explain why performance of both groups did not differ in the seesaw, as we did not manage to significantly change the task's appearance, a requisite that transfer tasks must fulfil (Seed et al., 2006).

To address this concern, we presented the birds with a second transfer task. To amend the limitation of the seesaw, we incorporated two major distinctions to make this task unique and completely novel for all birds: (1) the tool–reward interaction was direct (i.e. the tool was applied directly onto the reward without mediation of a target), and (2) the tool was already pre‐inserted in the apparatus (i.e. not requiring the individual to apply any motor action other than simply picking a hook). We found that birds who had received in phase 1 more diverse learning and motor experiences on multiple usages of the hooked tool (test group) had a higher solving accuracy (i.e. probability to solve a trial), needed less time to solve (i.e. in terms of latency to solve a trial and lower number of sessions to reach criterion altogether) and had a higher proportion of successful individuals in the cane task. These results provide support in favour of the prediction that the test birds would be better able to apply the hooking capacity of the tool to a task that did not resemble any other known task and that required a new solving principle with which none of the groups had experience. The fact that there were no group differences in the average latency to solve a trial in session 1 seems to indicate that there was no strong involvement of the motor component to find a solution. Hence, this two‐choice task allowed us to investigate subject's sensitivity to the functional spatial relationship between a reward and the tool used to obtain it (Teschke et al., 2011). Transfer in this context, at the very least, suggests that by having experienced a broader range of contingencies with the tool, the test group acquired a more general knowledge about the tool's affordances and function/s (including, but not limited to, a set of transferable skills), which varied in form and content from that acquired by the control group (Kamil, 1988; Tebbich et al., 2016). This may, in turn, have enabled the test group to grasp the new tool–reward interaction faster and to improve their problem‐solving competency. Therefore, the test group would have benefited from a broader knowledge with the potential to influence behaviour in various situations (i.e. be transferred and lead to the discovery and exploitation of adjacent opportunities) and to consequently increase behavioural flexibility (Anderson, 1976; Dienes & Perner, 1999; Tebbich et al., 2016).

A crucial difference between the test and control groups in phase 1 was that the test group was given the opportunity to practice how to use the tool in three different ways (i.e. to reach a target in three differently operated apparatuses). This, in turn, may have (a) strengthened the test birds' understanding of the complexity of the tool–target interactions and about how to correctly orientate the tool in relation to their own body in each apparatus (Fragaszy & Mangalam, 2018), and (b) assisted the development of a less rigid sensorimotor competence (i.e. not bound to a single tool‐use instance). However, even if the latter was the case, the test group would not have had a notorious advantage in the cane task, where the contribution of the motor component is considered practically neutralized, as birds did not have to manipulate the tool. Hence, no skilful manoeuvring or handling of the tool was required except for slightly pulling it, making both groups equal with respect to the influence of motor dexterity. For this reason, we do not think that enhanced sensorimotor competence played a significant driving role in the test group's performance on the cane task. We rather suggest that group differences might be attributable to differences in the knowledge acquired or potentiated during phase 1. Whether the cognitive mechanisms underlying these observed differences are based on associative learning and/or causal understanding is something that our data do not allow to determine, and a dichotomous vision that we do not endorse. Instead, we agree with previous research suggesting that they rather result from a non‐exclusive and mutually influencing combination of both processes (Tebbich et al., 2016; Teschke et al., 2013).

In contrast to the performance of the test group, the control group—which had an intensive experience with the tool in a single context—appeared less flexible to switch its use when faced with the cane task. Although they did transfer, they showed a lower probability of solving and a slower speed in doing so. At the proximate level, we speculate that this pattern could be due to the control group having had access to a narrower information of the tool's multi‐versatility and its potential applicability, which was intentionally limited during phase 1. This could have resulted in (a) a lack of knowledge about the relations between the problem elements, or (b) that such knowledge was coupled to a specific problem (Martin‐Ordas et al., 2008), namely the basket task, and hence less generalizable (Tebbich et al., 2016). Furthermore, studies in macaques indicate that tool use creates mental representations (Iriki et al., 1996; Maravita & Iriki, 2004) which tend to be rigidly used in relation to the behaviour in which they were originated (Macellini et al., 2012), leaving little space for adjustments and eventually leading to inflexibility. Our results suggest that control birds' acquisition of a less plastic substrate to fine‐tune performance might have led them to form a single representation of the tool (i.e. as a mean to hoist an out‐of‐reach reward in the vertical axis) by which they got fixated, as opposed to the three‐way representation formed by the test group. Eventually, the nature of our training might have driven subjects in the control group towards becoming more prone to suffer from functional fixedness, which needed to be overcome in order to approach the new problem efficiently (Fujita et al., 2011): by being intensively trained in the basket task (and repeating the corresponding behaviour in the seesaw task, where the hook was operated in a similar way), they might have been hindered to discover a new solution for the cane task different from the one that worked out for the basket and the seesaw (Duncker, 1945; Ebel et al., 2021; Harrison & Whiten, 2018). Despite the cane being perceptually and functionally dissimilar from the basket, this pattern of results could have emerged from the (over)training itself inducing proclivity to fixation in the control group.

Goffin's cockatoos' ability to gain different degrees of information about a tool and its purpose depending on the tool‐use training provided, and to use this information to tackle new problems more efficiently is a faculty that has been already observed in other bird taxa. Teschke et al. (2011), using a version of the cane task that involved several experimental conditions, found that woodpecker finches (WPF; *Cactospiza pallida*) and small tree finches (STF; *Camarhynchus parvulus*) were equally fast at solving the transfer task and showed similar improvement over the sequence of trials, with no difference between tool‐using and non‐tool‐using WPF, although STF outperformed non‐tool‐using WPF in success probability. In rooks (*Corvus frugilegus*; a non‐tool‐using species), Seed et al. (2006) tested eight subjects in a series of experiments with the modified two‐trap, a transfer task similar to our cane task in the sense that the tool was already placed inside the tube. In Experiment 2, birds that had solved configuration A of the tube in Experiment 1 were tested on configuration B, and vice versa, to assess whether they could transfer (which authors defined as “responding significantly correctly within the first 20 trials”). All rooks that had learnt the initial two‐trap task configuration (7 out of 8 subjects) were able to transfer in the new configuration. In Experiment 3, where subjects could not solve the task by using arbitrary cues or procedural rules based on the task configuration, all birds but one failed to transfer (which was defined as ≥9/10 correct trials or ≥15/20 in two 10‐trial blocks). Hence, our results provide the first evidence that parrots can, at the very least, parallel corvids' performance in transfer tasks.

Altogether, our results highlight that the ability to transfer poses a clear advantage in offsetting the downsides of innovating (Auersperg et al., 2017; Klump et al., 2015), as it can enable individuals to reach or create novel opportunities while reducing the cognitive and ecological resources required (e.g. time investment for manufacturing a new tool for a new purpose and mastering its use). To unveil *how* exactly transfer operates in Goffin's cockatoos, we encourage upcoming studies to use tasks based on the principle of triangulation (Heyes, 1993), such as sequential transfer tasks, where arbitrary features are removed successively from the original problem (see Taylor, Hunt, et al., 2009) for a reliable identification of the contribution of each component in the transfer process (e.g. determining the precise role of the motor component). Future studies should also aim to include transfer tasks where the type of tool is unattached and freely manipulable and the type of action applied involves a dynamic interaction, orientable by the agent (Fragaszy & Mangalam, 2018), which was the case in our seesaw task but not in the cane task. A final consideration concerns the fact that age was not accounted for in our study. Although ideally the eldest birds should not have all been part of the test group, the average age did not differ across groups. We hence consider that this factor might have had a minimal impact on our results, as Goffin's age does not necessarily translate into how much experimental experience they have (since they all began participating in tool use experiments in the same year), and none of the subjects were considered juvenile or senile. However, we would like to make a remark on the role that experience (defined by the STRANGE framework as “the opportunities for individual learning, such as participation in earlier experiments”; Webster & Rutz, 2020) can play on behaviour, as long‐lived subjects often accumulate experimental histories that could influence their test performance. To amend this and other potential sampling biases that may limit the generalisability of our findings (e.g. social background, genetic make‐up or acclimation), ideally a study of similar characteristics (using a different type of tool) should be conducted, where the subjects formerly allocated to the control group are the test subjects, and vice versa. This becomes however challenging in the case of the Goffins, given the reduced number of captive individuals available, and it (a) warrants cautious interpretation of our findings due to our relatively low sample size and (b) begs consideration for replicating the study in wild populations to assess the ecological relevance of this behaviour. Nevertheless, we believe that our study adds up to the growing catalogue of aptitudes of this species and expands our understanding of parrot cognition, opening interesting paths for future research in this field.

## AUTHOR CONTRIBUTIONS

**Paula Ibáñez de Aldecoa:** Conceptualization; investigation; writing – original draft; methodology; validation; visualization; formal analysis; software; project administration; data curation. **Alice M. I. Auersperg:** Conceptualization; investigation; funding acquisition; methodology; validation; project administration; supervision; resources. **Andrea S. Griffin:** Conceptualization; investigation; methodology; validation; project administration; supervision. **Sabine Tebbich:** Conceptualization; investigation; funding acquisition; methodology; validation; project administration; supervision; resources.

## CONFLICT OF INTEREST

The authors declare no potential conflicts of interest with respect to the research, authorship and/or publication of this article.

## CODE AVAILABILITY

The code for the statistical analysis is available from the corresponding author upon request.

## Supporting information


Appendix S1.

## Data Availability

The data sets generated and/or analysed during the current study are available at https://phaidra.univie.ac.at/view/o:1620423
