# Supplementary material for: Ratcheting up tool innovation in Goffin's cockatoos (Cacatua goffiniana): The effect of contextually diverse prior experience
Source: Ethology. 2022 Dec 19;129(3):133–45. doi: 10.1111/eth.13351 (PMC10108129; doi:10.1111/eth.13351)
Supplement: Supplementary file 1 — Appendix S1. [file ETH-129-133-s001.docx]

**Ratcheting up tool innovation in Goffin’s cockatoos (*Cacatua goffiniana*): The effect of contextually diverse prior experience**

Paula Ibáñez de Aldecoa^1^, Alice M. I. Auersperg^2^, Andrea S. Griffin^3^ and Sabine Tebbich^1^

^1^Department of Behavioural and Cognitive Biology, University of Vienna, Vienna 1030, Austria

^2^Messerli Research Institute, University of Veterinary Medicine Vienna, University of Vienna, Medical University of Vienna, Austria

^3^School of Psychology, University of Newcastle, Callaghan, New South Wales 2308, Australia

**Author Information**

Paula Ibáñez de Aldecoa: <https://orcid.org/0000-0003-3858-5107>

Alice M. I. Auersperg: <https://orcid.org/0000-0001-7405-9791>

Andrea S. Griffin: <https://orcid.org/0000-0003-4624-9904>

Sabine Tebbich: <https://orcid.org/0000-0003-3971-2139>

Correspondence concerning this article should be addressed to Paula Ibáñez de Aldecoa, Department of Behavioural and Cognitive Biology, Biologiezentrum University of Vienna. Schlachthausgasse 43, 1030 Vienna, Austria. E-mail: [paula.i.aldecoa@univie.ac.at](mailto:paula.i.aldecoa@univie.ac.at)

**SUPPLEMENTARY MATERIALS**

**A) Subject information**

**Table S1**. **Names, division into two testing groups, sex and hatching year of the 13 subjects who participated in this study**.

| Group | Name | Sex | Hatched |
| --- | --- | --- | --- |
| Test | Dolittle  Figaro  Fini  Heidi  Mayday  Muppet  Pipin | male  male  female  female  female  male  male | 2011  2007  2007  2010  2011  2010  2008 |
| Control | Kiwi  Konrad  Moneypenny  Muki  Olympia  Zozo | male  male  female  male  female  male | 2010  2010  2010  2011  2010  2010 |

*Housing conditions*

Subjects were permanently kept in a social group in a large aviary with indoor and outdoor area (indoors: 45m^2^ ground space, 3 to 6 m high; outdoors: 150m^2^, 3 to 4,5m high). The indoor area is kept at 20°C during wintertime and electric UV light is switched automatically at 12:12 hours light-dark cycles. Food (Cardi-seed mix by www.animalexperts.at supplemented with dried fruit and vitamins; diverse sorts of fresh fruits and fresh and cooked vegetables, soy yoghurt), mineral sources and fresh drinking water was available *ad libitum*. Special treats such as nuts were only offered as food rewards during experimental routines. Enrichment consisted of a variety of perches, trees, parrot toys, cardboard and bathing opportunities. All birds were hand-raised and marked with colored foot rings for identification.

**B) Supplementary results. Tables and figures**

**Table S2**. **Number of solved trials per session and mean across all sessions for each subject in the cane task**. Legend: (T) = test group; (C): control group; S: session number (i.e., S1 = session 1, et cetera); (-): sessions that did not take place (i.e., individual had already reached criterion); (*): individuals who did not reach criterion (Muki, Olympia and Moneypenny). Note: Number in bold corresponds to session when each individual reached solving criterion (80% of correct trials in two consecutive sessions)

| Subject | S1 | S2 | S3 | S4 | S5 | S6 | S7 | S8 | S9 | S10 | Mean | |
| --- | --- | --- | --- | --- | --- | --- | --- | --- | --- | --- | --- | --- |
| (T) Dolittle | 6 | 7 | **11** | **10** | - | - | - | - | - | - | | 8.5 |
| (T) Muppet | 7 | 8 | 10 | 9 | **10** | **11** | - | - | - | - | | 9.2 |
| (T) Figaro | 7 | 6 | 9 | 9 | **10** | **10** | - | - | - | - | | 8.5 |
| (T) Fini | 4 | 6 | **11** | **10** | - | - | - | - | - | - | | 7.8 |
| (T) Mayday | 6 | 9 | 10 | 9 | 9 | **11** | **10** | - | - | - | | 9.1 |
| (T) Heidi | 7 | 9 | 9 | 9 | 9 | **10** | **10** | - | - | - | | 9 |
| (T) Pipin | 5 | 8 | 9 | **10** | **10** | - | - | - | - | - | | 8. |
| (C) Muki* | 4 | 5 | 6 | 7 | 7 | 8 | 8 | 9 | 9 | 8 | | 7.1 |
| (C) Olympia* | 4 | 5 | 5 | 6 | 7 | 7 | 8 | 9 | 8 | 8 | | 6.7 |
| (C) Moneypenny* | 6 | 7 | 7 | 8 | 8 | 9 | 9 | 10 | 9 | 9 | | 8.2 |
| (C) Kiwi | 6 | 8 | 7 | 8 | 9 | **10** | **11** | - | - | - | | 8.4 |
| (C) Konrad | 7 | 6 | 7 | 7 | 8 | 8 | 9 | **10** | **10** | - | | 8 |
| (C) Zozo | 7 | 7 | 7 | 7 | 8 | 8 | **10** | **11** | - | - | | 8.1 |

**Table S3**. **Number of solved trials per session and mean across all sessions for each subject in the seesaw task**. Legend: (T) = test group; (C): control group; S: session number (i.e., S1 = session 1, et cetera); (-): sessions that did not take place (i.e., individual had already reached criterion); (*): individuals who did not reach criterion (Muki and Olympia). Note: Number in bold corresponds to session when each individual reached solving criterion (80% of correct trials in one session).

| Subject | S1 | S2 | S3 | Mean |
| --- | --- | --- | --- | --- |
| (T) Dolittle | 9 | **10** | - | 9.5 |
| (T) Muppet | **12** | - | - | 12 |
| (T) Figaro | **12** | - | - | 12 |
| (T) Fini | **11** | - | - | 11 |
| (T) Mayday | 7 | 8 | **12** | 9 |
| (T) Heidi | 6 | 9 | **10** | 8.3 |
| (T) Pipin | 9 | **12** | - | 10.5 |
| (C) Muki* | 2 | 3 | 4 | 2.7 |
| (C) Olympia* | 2 | 6 | 5 | 3.4 |
| (C) Moneypenny | 8 | 8 | **10** | 8.6 |
| (C) Kiwi | **12** | - | - | 12 |
| (C) Konrad | **10** | - | - | 10 |
| (C) Zozo | **11** | - | - | 11 |

**Table S4**. **Table of coefficients for the fixed effects on seesaw task (model 1)**. Legend: nr.: number; (:): interaction term; SE: standard error; CI: 95% confidence intervals; (-). Note: Reference values for *group* and *sex* are “control” and “male”, respectively. *Session number* was z-transformed. original mean ± standard deviation of session number was 2 ± 0.82.

|  | Estimate | SE | z-value | p-value | Lower CI | Upper CI |
| --- | --- | --- | --- | --- | --- | --- |
| Intercept | 1.664 | 0.747 | 2.227 | 0.026 | 0.249 | 3.502 |
| Group | 1.494 | 0.883 | 1.692 | 0.091 | -0.383 | 3.534 |
| Session nr. | 0.300 | 0.211 | 1.425 | 0.154 | -0.106 | 0.725 |
| Sex | -1.651 | 0.910 | -1.814 | 0.069 | -3.876 | 0.133 |
| Group:Session nr. | 0.417 | 0.339 | 1.229 | 0.219 | -0.238 | 1.101 |

**Table S5**. **Number of sessions required by each subject of the test group to complete the training and test stages on phase 1 for each apparatus (basket, skate, trap)**. Legend: nr.: number.

| Subject | Basket  training | Basket test | Trap training | Trap test | Skate training | Skate  test | Total nr. sessions |
| --- | --- | --- | --- | --- | --- | --- | --- |
| Dolittle | 1 | 2 | 6 | 4 | 4 | 2 | 19 |
| Muppet | 3 | 2 | 6 | 3 | 4 | 2 | 20 |
| Figaro | 1 | 2 | 5 | 3 | 2 | 2 | 15 |
| Fini | 1 | 2 | 6 | 2 | 6 | 2 | 19 |
| Mayday | 2 | 2 | 5 | 2 | 5 | 4 | 20 |
| Heidi | 3 | 2 | 5 | 3 | 6 | 2 | 21 |
| Pipin | 4 | 2 | 5 | 2 | 4 | 3 | 20 |

**Table S6**. **Number of sessions required by each subject of the control group to complete the training and test stages on phase 1 (task: basket)**. Legend: nr.: number. Note: The number between brackets in the column ‘basket testing’ indicates the extra sessions given to each subject once it reached criterion, to achieve a total number of sessions comparable to the test group.

| Subject | Basket training | Basket testing  (+ extra time) | Total nr.  sessions |
| --- | --- | --- | --- |
| Muki | 8 | 4 (+ 8 Extra) | 20 |
| Olympia | 7 | 7 (+ 7 Extra) | 21 |
| Moneypenny | 1 | 3 (+ 16 Extra) | 20 |
| Kiwi | 2 | 2 (+ 14 Extra) | 18 |
| Konrad | 2 | 3 (+ 13 Extra) | 18 |
| Zozo | 2 | 3 (+ 12 Extra) | 17 |

**Table S7: Independent *t*-tests for: (a) the number of sessions required in the cane task; (b) the number of sessions required in the seesaw task; (c) the trials solved in the cane task; (d) the trials solved in the seesaw task.** Legend: (*) Significant *p*-values at *α* = 0.05 (shown in bold). Abbreviations: Ctrl = control group; N = sample size; SD = standard deviation; CI = 95% confidence intervals.

| T-test | N | Mean ± SD | p-value | t-value | Lower CI | Upper CI |
| --- | --- | --- | --- | --- | --- | --- |
| (a) | Test: 7  Ctrl: 6 | 5.57 ± 1.27  9 ± 1.26 | **0.001*** | -4.227 | -5.657 | -1.913 |
| (b) | Test: 7  Ctrl: 6 | 1.85 ± 0.89  2 ± 1.1 | 0.092 | -1.869 | -2.255 | 0.202 |
| (c) | Test: 7  Ctrl: 6 | 8.42 ± 0.45  7.76 ± 0.75 | **0.024*** | 2.645 | 0.161 | 1.805 |
| (d) | Test: 7  Ctrl: 6 | 11 ± 1.41  11 ± 1 | 0.577 | -0.582 | -2.595 | 1.556 |

**Table S8: Table of coefficients for the fixed effects of the Cox regression for the seesaw task (model 3)**. Legend: nr.: number; (:): interaction term; (*) Significant *p*-values at *α* = 0.05 are shown in bold; Coef.: coefficients; Exp.: exponentiated coefficients; SE: standard error; CI: 95% confidence intervals. Note: Reference values for *group* and *sex* are “control” and “male”, respectively; *Session number* and *trial number* were z-transformed; original mean ± standard deviation = 2 ± 0.82 and 6.5 ± 3.45, respectively.

|  | Coef. | Exp. | SE | z-value | p-value | Lower CI | Upper CI |
| --- | --- | --- | --- | --- | --- | --- | --- |
| Group | 1.607 | 4.987 | 1.319 | 1.22 | 0.220 | -0.979 | 4.193 |
| Trial nr. | -0.574 | 0.563 | 0.412 | -1.39 | 0.160 | -1.383 | 0.235 |
| Session nr. | 1.607 | 4.991 | 0.730 | 2.2 | **0.028*** | 0.176 | 3.039 |
| Sex | -0.427 | 0.652 | 0.756 | -0.57 | 0.570 | -1.908 | 1.054 |
| Group:Trial nr.:Session nr. | 0.297 | 1.346 | 0.524 | 0.57 | 0.571 | -0.731 | 1.326 |

**Table S9**. **Independent *t*-tests for the number of correct trials in session 1 of (a) the cane task and (b) the seesaw task**. Legend: (*) Significant *p*-values at *α* = 0.05 (shown in bold). Abbreviations: Ctrl = control group; N = sample size; SD = standard deviation; CI = 95% confidence intervals.

| Task | N | Mean ± SD | p-value | t-value | Lower CI | Upper CI |
| --- | --- | --- | --- | --- | --- | --- |
| 1. Cane | Test: 7  Ctrl: 6 | 7 ± 0.57  4.83 ± 0.75 | **< 0.001*** | 6.248 | 1.422 | 3.193 |
| 1. Seesaw | Test: 7  Ctrl: 6 | 9.57 ± 2.37  7.5 ± 4.46 | 0.062 | 2.164 | -0.211 | 7.121 |

**Table S10**. **Independent *t*-tests for the average time to solve a trial (in seconds) in session 1 of (a) the cane task and (b) the seesaw task**. Legend: (*) Significant *p*-values at *α* = 0.05 (shown in bold). Abbreviations: Ctrl = control group; N = sample size; SD = standard deviation; CI = 95% confidence intervals.

| Task | N | Mean ± SD | p-value | t-value | Lower CI | Upper CI |
| --- | --- | --- | --- | --- | --- | --- |
| 1. Cane | Test:  Ctrl: | 6.26 ± 2.61  10.78 ± 6.5 | 0.351 | -0.992 | -10.069 | 4.046 |
| 1. Seesaw | Test:  Ctrl: | 67.13 ± 35.74  125.49 ± 99.35 | 0.479 | 0.758 | -72.585 | 135.439 |
